# Subtotal esophagectomy and concurrent reconstruction with free jejunal flap for primary esophageal cancer after pancreatoduodenectomy

**DOI:** 10.1186/s40792-024-01919-5

**Published:** 2024-05-22

**Authors:** Kazuya Moriwake, Kazuhiro Noma, Kento Kawasaki, Tasuku Matsumoto, Masashi Hashimoto, Takuya Kato, Naoaki Maeda, Shunsuke Tanabe, Yasuhiro Shirakawa, Toshiyoshi Fujiwara

**Affiliations:** 1 Department of Gastroenterological Surgery, Okayama University Graduate School of Medicine, Dentistry and Pharmaceutical Sciences, 2-5-1 Shikata-Cho, Kita-Ku, Okayama, 700-8558 Japan; 2 Department of Surgery, Hiroshima City Hiroshima Citizens Hospital, 7-33 Motomachi, Naka-Ku, Hiroshima, Japan

**Keywords:** Reconstruction with the free jejunum flap, Subtotal esophagectomy, After pancreatoduodenectomy

## Abstract

**Background:**

Pancreatoduodenectomy and subtotal esophagectomy are widely considered the most invasive and difficult surgical procedures in gastrointestinal surgery. Subtotal esophagectomy after pancreatoduodenectomy is expected to be extremely difficult due to complicated anatomical changes, and selecting an appropriate intestinal reconstruction method will also be a difficult task. Therefore, perhaps because the method is considered impossible, there have been few reports of subtotal esophagectomy after pancreatoduodenectomy.

**Case presentation:**

A 73-year-old man with a history of pancreatoduodenectomy was diagnosed with superficial thoracic esophageal squamous cell carcinoma. Definitive chemoradiation therapy was recommended at another hospital; however, he visited our department to undergo surgery. We performed the robot-assisted thoracoscopic subtotal esophagectomy. There were some difficulties with the reconstruction: the gastric tube could not be used, the reconstruction was long, and the organs reconstructed in the previous surgery had to be preserved. However, the concurrent reconstruction was achieved with the help of a free jejunal flap and vascular reconstruction. All reconstructions from the previous surgery, including the remnant stomach, were preserved via regional abdominal lymph node dissection. After reconstruction, intravenous indocyanine green showed that circulation in the reconstructed intestines was preserved. On postoperative day 1, no recurrent nerve paralysis was observed during laryngoscopy. The patient could start oral intake smoothly 2 weeks after surgery and did not exhibit any postoperative complications related to the reconstruction. The patient was transferred to another hospital on postoperative day 21.

**Conclusions:**

Owing to the free jejunal flap interposition method, we safely performed one stage subtotal esophagectomy and concurrent reconstruction, preservation of the remnant stomach, and pancreaticobiliary reconstruction in patients with a history of pancreatoduodenectomy. We believe that this method is acceptable and useful for patients undergoing complicated reconstruction.

## Background

Pancreatoduodenectomy (PD) and subtotal esophagectomy are the most invasive and difficult procedures in gastroenterological surgery. When both procedures are performed in one patient, it is expected to be particularly difficult to reconstruct the gastrointestinal tract due to anatomical changes and to preserve the circulation of the reconstructed organs. Definitive chemoradiation therapy (CRT) is an acceptable alternative therapy to surgery for primary esophageal cancer [1]; therefore, it can be selected, especially after surgery with complicated reconstruction, such as PD. Although there have been several case reports of synchronous esophagectomy and PD [2–4], PD after esophagectomy [5, 6], and partial esophagectomy for non-esophageal cancer after PD [7]. Although there is only one published report on subtotal esophagectomy after PD, in that case, reconstruction was performed in the second stage operation [8]. There are no reports that one stage subtotal esophagectomy and reconstruction while preserving the pancreaticobiliary reconstruction for post-PD esophageal cancer. Here, we report a case of primary esophageal cancer in a patient who had previously undergone PD. We performed a robot-assisted thoracoscopic subtotal esophagectomy and concurrent reconstruction with free jejunal flap interposition.

## Case presentation

**Fig. 1:**
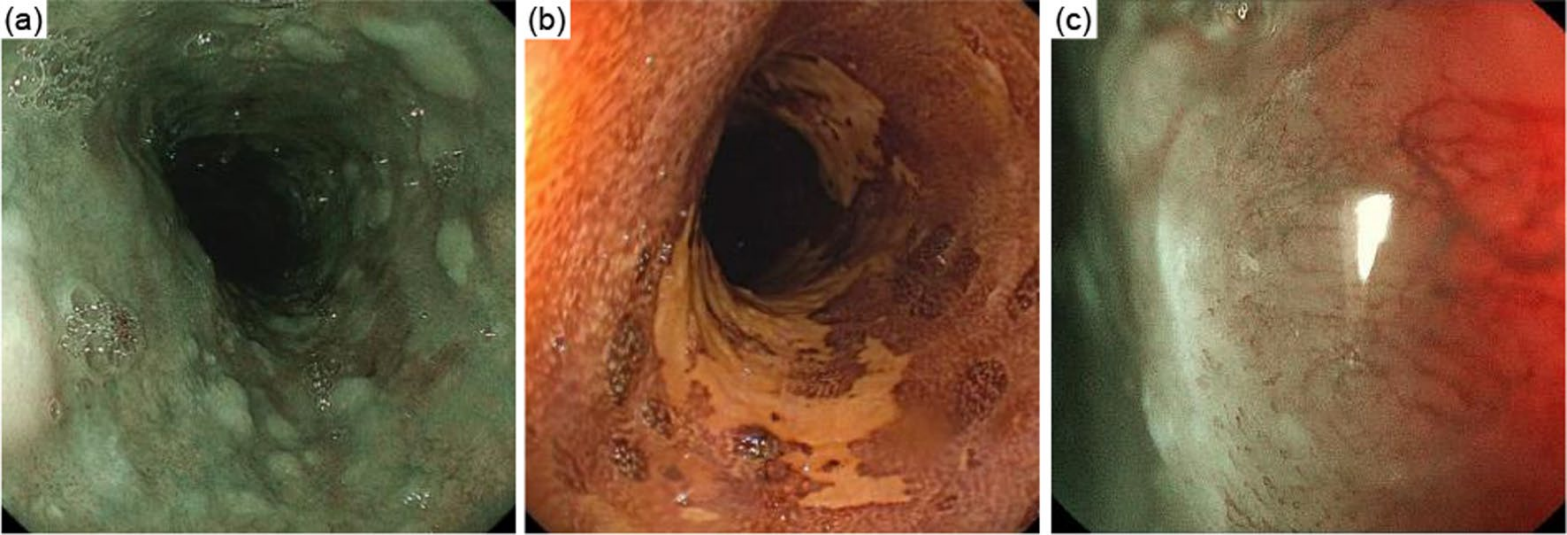
Findings of the endoscopy. **a** Full circumferential 0–IIc + 0–IIa, described as a brownish area via narrow band imaging in the upper thoracic esophagus. **b** The lesion was described as iodine-unstained area. **c** A magnified view showing the B2 and B3 vessels

**Fig. 2:**
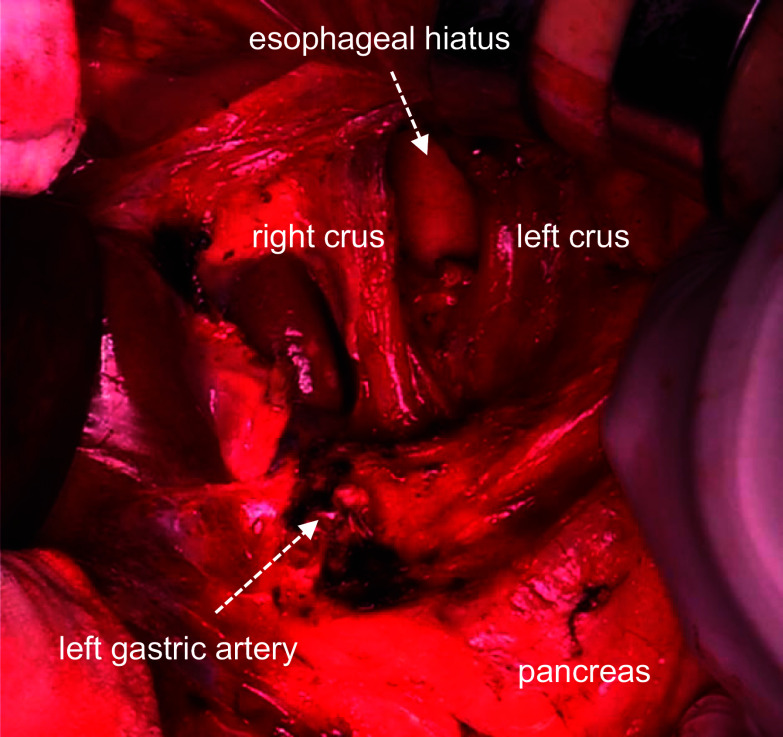
After lymph node dissection of abdominal region. The abdominal lymph nodes (no. 1, 2, 3a, 7, 9, 19, and 20) were dissected

**Fig. 3:**
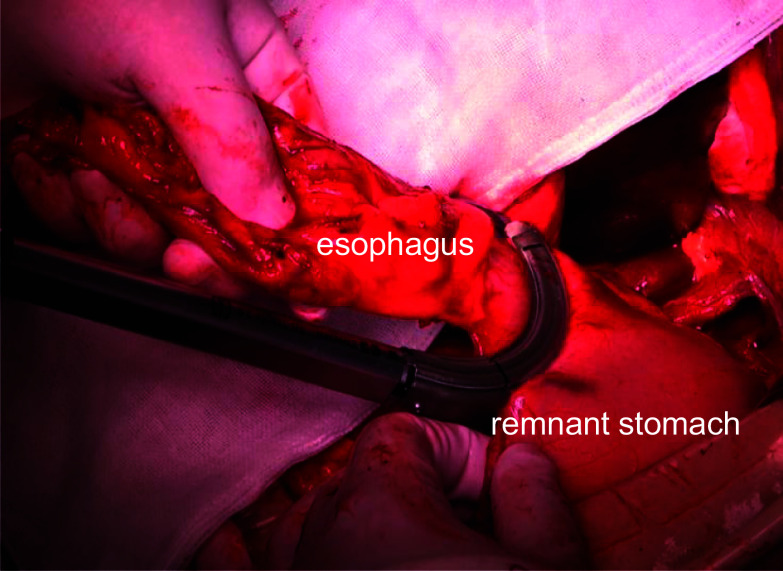
Subtotal esophagectomy preserving almost all of the remnant stomach

**Fig. 4:**
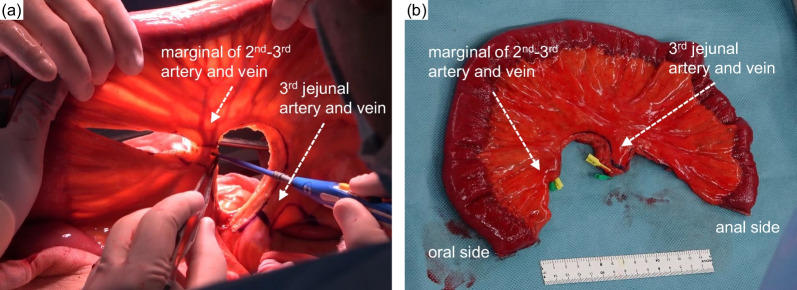
Free jejunal flap for reconstruction. **a** The margins of the second and third jejunal arteries were used for reconstruction. **b** The free jejunal flap

**Fig. 5:**
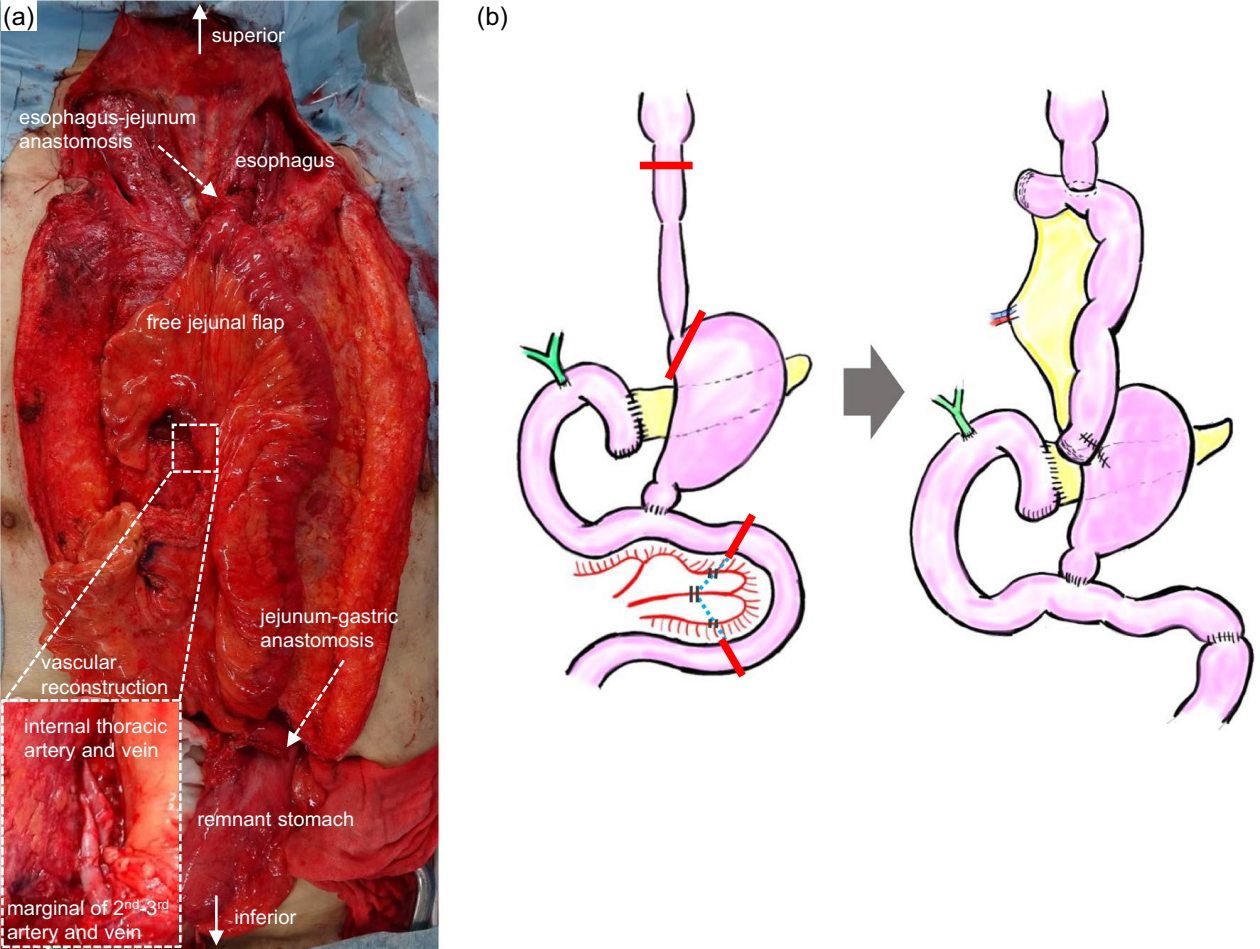
Intestinal and vascular reconstruction in the subcutaneous route. **a** The picture and **b** the schema of reconstruction

A 73-year-old man was diagnosed with upper thoracic esophageal squamous cell carcinoma based on endoscopic findings at another clinic (Fig. 1a–c). It showed B2 vessels: abnormal vessels with poor loop formation and B3 vessels: markedly dilated abnormal vessels which suggest submucosal invasion based on Japan Esophageal Society classification. He had undergone pylorus-preserving PD (PPPD) with Child’s reconstruction for a mixed-type intraductal papillary mucinous neoplasm 3 years earlier at the previous hospital and was referred to the department of surgery of the same hospital. However, although the surgeons there recommended definitive CRT due to difficulty in performing subtotal esophagectomy after PD, the desired to undergo the surgical treatment and was thus referred to our department. The clinical diagnosis was upper thoracic esophageal squamous cell carcinoma, cT1bN0M0, cStage I, UICC 8th [9]. The planned surgical procedure was robot-assisted thoracoscopic subtotal esophagectomy and lymph node dissection in three fields (neck, thorax, and abdomen) with the reconstruction of the free jejunal interposition. The surgery was performed as planned. For prone thoracic manipulation, robot-assisted thoracic lymph node dissection and thoracic esophageal resection were performed at the level of the aortic arch. The thoracic duct and bilateral recurrent nerves were preserved using a nerve integrity monitor (NIM, Medtronic, Tokyo, Japan). Subsequently, cervical and abdominal manipulations were performed in the supine position. For neck manipulation, bilateral recurrent nerve sparing, lymph node dissection, and cervical esophagus resection were performed. During abdominal manipulation with a median incision, there were no significant peritoneal adhesions. The lesser curvature of the remnant stomach was dissected and the left gastric artery was resected. Standard regional abdominal lymph node dissection for thoracic esophageal cancer was performed, while preserving the left gastroepiploic, short gastric, and posterior gastric arteries (Fig. 2). The stomach was cut using an automated suture device just below the esophagogastric junction and the specimen was removed, preserving almost the entire remnant stomach (Fig. 3). After observing the vessels of the jejunum in detail, the main trunks of the first and second jejunal arteries were used for the reconstruction of the previous PPPD. Therefore, a free jejunal flap with the margins of the second and third jejunal arteries was used for the reconstruction (Fig. 4a, b). Subcutaneous reconstruction was performed, and the right internal thoracic artery and vein were used for vascular anastomosis (Fig. 5a, b). The anastomosis of the cervical esophagus and free jejunum flap was performed using an automated anastomosis device for end-to-side anastomosis. The anastomosis of the free jejunum flap and remnant stomach was performed using an automated suture device. Finally, an evaluation of the circulation using intravenous indocyanine green (ICG) revealed that the circulation of the reconstructed organ was preserved. On postoperative day 1, no recurrent nerve paralysis was shown by the laryngeal fiber. The patient was discharged from the intensive care unit on postoperative day 6 and started oral intake on postoperative day 14. The patient had no postoperative complications related to the reconstruction and was transferred to another hospital on postoperative day 21 for rehabilitation. The lymph node metastasis was observed in 106recR in the postoperative pathological findings. The pathological result indicated esophageal squamous cell carcinoma, pT1bN1M0, pStageIIB, UICC 8th [9].

## Discussion

In our patient with a history of PPPD, subtotal esophagectomy and concurrent reconstruction were performed to preserve the remnant stomach and pancreaticobiliary reconstruction. Owing to the anticipated intra-abdominal adhesions and the need to confirm the jejunal vessels in detail, an open abdominal procedure was performed. To ensure preservation of the circulation of the remnant stomach, the left gastroepiploic artery, short gastric artery, and posterior gastric artery were preserved, while abdominal lymph node dissection was performed as usual for thoracic esophageal cancer. In addition, to preserve the circulation of the reconstructed jejunum of PPPD, we considered that the main trunks of the first and second jejunal arteries were involved in pancreaticobiliary reconstruction in the previous surgery; therefore, a free jejunal flap was obtained from the jejunal artery on the peripheral side and used for reconstruction. Intravenous indocyanine green was used to confirm the preservation of the circulation of the reconstructed organs.

We selected subtotal esophagectomy and concurrent reconstruction as the initial treatment in this case, despite certain difficulties. The esophageal cancer practice guidelines 2022 by the Japan esophageal society recommend esophagectomy for cStage I thoracic esophageal cancer unless esophageal preservation is desired [1]. Radical CRT is an alternative treatment to surgery for esophageal cancer; however, there are few case reports of patients who have undergone subtotal esophagectomy after PD, which is expected to be more difficult, as in this case. Although a recent report showed that radical CRT is not inferior to surgery for stage 0 or I esophageal squamous cell carcinoma [10], a detailed analysis suggested that CRT is indeed inferior to surgery in terms of 5-year long-term recurrence-free survival. In such a case, if recurrence is observed after CRT, there is a risk that the vessels for vascular reconstruction of the free jejunum, for example internal thoracic artery, may be included in the irradiated area and reconstruction may not be possible at salvage surgery.

In our case, intestinal reconstruction was performed using a free jejunal flap. There were several reasons for this. First, we frequently perform reconstructions with a free jejunal flap as usual. In the case had undergone PPPD, gastric tube reconstruction, which is most frequently performed in our hospital, could not be performed. At our institution, in collaboration with otolaryngology and plastic surgery departments, we actively perform reconstruction using free jejunum in pharyngolaryngectomy for hypopharyngeal and laryngeal cancer. The second reason was the distance of the reconstruction to preserve the remnant stomach and jejunum involved in the previous pancreaticobiliary reconstructions. In previous reports, esophagectomies in patients with previous PD were reconstructed using a pedicled transverse colon [7]. Because of the resection range of the esophagus, lower esophagus, the intestinal length needed for reconstruction was short. In contrast, in our case, it was necessary to preserve the remnant stomach and jejunum used for the reconstruction of the previous PPPD, and the reconstruction distance between the cervical esophagus and the remnant stomach was long. Third, we preferred straight reconstructive intestinal tract. In another report, reconstruction with a pedicled jejunum in subtotal esophagectomy for esophageal cancer was performed [8]. There is a concern that reconstruction with a pedicled jejunum or pedicled colon may result in meandering of the reconstructed intestinal tract, leading to dysphagia, in some case intestinal obstruction, and decreased food intake. Adjusting the length of the jejunum, we could prevent the intestinal tract from sagging due to gravity and maintain the reconstructed jejunum in a straight position. Therefore, we determined that it is possible to safely perform reconstruction with interposition of free jejunum without affecting the jejunal reconstruction in previous SSPPD, while maintaining patient’s quality of life, and selected this method for reconstruction. We highlight the significance of this case report as the first reported case of performing one stage subtotal esophagectomy and reconstruction while preserving the pancreaticobiliary reconstruction for post-pancreaticoduodenectomy esophageal cancer. Our study observations suggest that reconstruction with free jejunal interposition may be useful in cases of subtotal esophagectomy with complicated anatomical variations after PD.

## Conclusions

A patient with thoracic esophageal cancer and history of PPPD underwent subtotal esophagectomy. Using free jejunal interposition, we safely performed concurrent reconstruction while preserving the remnant stomach and pancreaticobiliary reconstructions.

## Data Availability

Data sharing is not applicable to this article, as no datasets were generated or analyzed in the current study.

## Abbreviations

PD: Pancreatoduodenectomy
PPPD: Pylorus-preserving pancreatoduodenectomy
CRT: Chemoradiation therapy
